# Pre- and post-cranioplasty hydrocephalus in patients following decompressive craniectomy for ischemic stroke: a systematic review and meta-analysis

**DOI:** 10.1007/s10143-025-03650-7

**Published:** 2025-06-18

**Authors:** I. Khalil, R. Sayad, S. K. Kamal, Z. Hussein, S. Allam, A. L. F. Caprara, J. P. Rissardo

**Affiliations:** 1 Faculty of Medicine, Alexandria University, Alexandria, 5372066 Egypt; 2 Faculty of Medicine, Assiut University, Assiut, 71515 Egypt; 3 Faculty of Medicine, Cairo University, Cairo, Egypt; 4 Faculty of Medicine, Minia University, Minia, Egypt; 5 Faculty of Medicine, Galala University, Suez, Egypt; 6 Neurology Department, Cooper University Hospital, Camden, NJ 08103 USA

**Keywords:** Hydrocephalus, Cranioplasty, Decompressive hemicraniectomy, Ischemic stroke, Shunt

## Abstract

Decompressive hemicraniectomy (DC) for malignant ischemic stroke can lead to hydrocephalus (HC). The dynamics of hydrocephalus at the time of subsequent cranioplasty (CP) are not well characterized. We aimed to systematically review and meta-analyze the rates of hydrocephalus and ventriculomegaly before and after CP, shunt dependency, and hydrocephalus resolution in patients who underwent DC for ischemic stroke. Following the PRISMA guidelines, we searched PubMed, Embase, Scopus, Web of Science, and Cochrane Library through January 2025 (registration number CRD420251039185). in studies reporting hydrocephalus in patients undergoing DC followed by CP for ischemic stroke. Inclusion criteria were ≥ 10 patients in the English language. Data on hydrocephalus/ventriculomegaly rates (pre-CP and post-CP), shunt dependency, and resolution were extracted. Study quality was assessed using the MINORS criteria. Random-effects meta-analyses were performed to calculate pooled proportions with 95% confidence intervals (CI). Heterogeneity was assessed using I², and sources were explored using meta-regression and sensitivity analyses. Ten retrospective studies, involving 579 patients, met the inclusion criteria. The study quality was predominantly fair (6/10) or low (3/10). Pooled rates were: pre-CP hydrocephalus 0.40 (95% CI: 0.17–0.65; I²=95.5%), post-CP hydrocephalus 0.46 (0.14–0.79; I²=89.8%), pre-CP ventriculomegaly 0.43 (0.21–0.67; I²=95.5%), and post-CP ventriculomegaly 0.46 (0.14–0.79; I²=89.8%). There was no significant difference between the pre- and post-CP hydrocephalus rates (*P* = 0.6481). The pooled rate of shunt-dependent hydrocephalus was 0.11 (0.04–0.22; I²=61.4%), and the hydrocephalus resolution rate post-CP was 0.27 (0.07–0.53; I²=68.7%). Significant heterogeneity was observed across most outcomes, and a potential publication bias was detected. The GRADE assessment indicated very low-to low-quality evidence. Radiographic hydrocephalus or ventriculomegaly is common (approximately 40–46%) both before and after cranioplasty in patients treated with DC for ischemic stroke, with no significant change immediately post-CP identified in this analysis. However, clinically significant hydrocephalus requiring shunting occurs less frequently (approximately 11%). The quality of evidence is limited by the retrospective study design and high heterogeneity. High-quality prospective studies with standardized definitions and follow-up are needed to better understand hydrocephalus dynamics and the impact of cranioplasty timing in this population.

**Supplementary Information:**

The online version contains supplementary material available at 10.1007/s10143-025-03650-7.

## Clinical trial number

Not applicable

## Introduction

Stroke is a leading cause of mortality worldwide. Epidemiological studies suggest that approximately one in four individuals over the age of 25 years will experience stroke in their lifetime. Notably, > 62% of these stroke events are ischemic [19]. In rare instances, large-vessel strokes, such as those affecting the Middle Cranial Artery (MCA) or Internal Cerebral Artery (ICA), can lead to severe cerebral edema and increased intracranial pressure. This condition, known as malignant MCA stroke, may be further complicated by cerebral herniation and hypoperfusion, potentially resulting in death if not promptly diagnosed and treated [4, 10, 12, 17]. Decompressive hemicraniectomy is an emerging therapy for selected cases of malignant MCA infarctions that involves the removal of a skull bone flap to alleviate pressure and enhance brain tissue oxygenation [4, 10, 12, 17]. A small systematic review and meta-analysis comparing mortality rates between medically and surgically treated patients revealed significantly lower mortality in those who underwent surgical treatment [4].

After the acute stage is treated with decompressive hemicraniectomy, cranioplasty is performed to restore the skull. CSF dynamics present a unique set of challenges related to cranioplasty, both pre- and post-operatively. Hydrocephalus is a known complication of DC for ischemic stroke [10, 12, 17, 18], although its evolution is less clearly established with studies reporting variable rates [14]. It remains debatable whether cranioplasty per se plays a role in the resolution of hydrocephalus, possibly by normalizing intracranial perfusion and CSF flow, or whether cranioplasty may aggravate any remaining CSF disturbances [14]. It is therefore important to understand the incidence of hydrocephalus both prior to and following this reconstructive surgery to guide patient management, shunt placement indications, and the timing of surgical intervention [10, 12].

However, such a procedure has the potential for several complications, such as hematomas, external cerebral herniation, CSF leak/fistulae, and seizures, which may affect clinical outcomes and quality of life [7]. Hydrocephalus following decompressive hemicraniectomy has been reported in several studies. In some cases, the original pathology may have contributed to the development of hydrocephalus [1, 18]. In others, such as MCA infarctions and large intracerebral hematomas, no association between the original pathology and hydrocephalus has been found [10, 12, 17]. Many studies have hypothesized an association between decompressive hemicraniectomy and the development of hydrocephalus [12].

Development of hydrocephalus following decompressive hemicraniectomy for ischemic stroke is a known complication, hypothesized to be partially related to changes in CSF absorption and compliance mechanisms secondary to a large cranial defect [10, 12, 14, 17]. Maintaining the cranial vault in a closed system is hypothesized  to reverse the effects of these altered cranial dynamics through a subsequent cranioplasty procedure. However, clinical observations and study outcomes are mixed, with some experiencing resolution of ventricular enlargement after cranioplasty, while others developing persistent or de novo hydrocephalus that requires shunting [12, 14, 18]. This variation highlights the necessity of systematically assessing the incidence and variations of hydrocephalus within the subpopulation of patients undergoing cranioplasty after DC for ischemic stroke.

Hydrocephalus is a known complication of decompressive hemicraniectomy for malignant MCA infarction, and determining its course is especially relevant when planning  consecutive cranioplasty. The optimal timing for cranioplasty and its effect on cerebrospinal fluid dynamics and the resolution or progression of hydrocephalus are topics of ongoing clinical research. The relationships observed between cranioplasty and rates of hydrocephalus and ventriculomegaly, as well as shunting, are important to clarify by quantifying the rates before and after cranioplasty to facilitate informed clinical practice.

## Methods

Our meta-analysis was conducted in accordance with the PRISMA (Preferred Reporting Items for Systematic Reviews and Meta-Analyses) guidelines. We employed a comprehensive statistical approach to analyze the hydrocephalus rates in patients undergoing cranioplasty [11]. The study protocol was prospectively registered in the International Prospective Register of Systematic Reviews (registration number CRD420251039185).

### Search strategy and study selection

We conducted a comprehensive search of electronic databases, such as PubMed, Embase, Scopus, Web of Science, and Cochrane Library, up to January 2025. The search terms are detailed in Supplementary File 1. Furthermore, we manually reviewed the reference lists of the selected studies and pertinent review articles to identify additional qualified studies. Two independent reviewers evaluated the titles and abstracts for eligibility based on the predefined inclusion criteria. Studies were considered if they (1) involved patients who had undergone decompressive craniectomy followed by cranioplasty, (2) provided data on hydrocephalus rates before and/or after cranioplasty, (3) included a minimum of 10 patients, and exclusion criteria included case reports, review articles, technical notes, conference abstracts, and studies lacking sufficient data for extraction. Disagreements were resolved by a third reviewer.

### Data extraction and quality assessment

Two reviewers independently extracted the data using a standardized form. Information collected included the first author, publication year, study design, sample size, patient demographics, timing of cranioplasty, rates of pre-cranioplasty hydrocephalus, post-cranioplasty hydrocephalus, shunt-dependent hydrocephalus, and hydrocephalus resolution. For studies reporting ventriculomegaly rather than hydrocephalus, we extracted these data separately.

### Quality assessment

Risk of bias in the included studies was assessed using the Methodological Index for Non-Randomized Studies (MINORS) criteria, a validated tool specifically designed for non-randomized interventional studies [16]. Two independent reviewers assessed each study, and discrepancies were resolved by consensus or by a third reviewer when necessary.

Based on cumulative scores, studies were categorized as extremely poor quality (0–4), low quality (5–7), fair quality (8–12), and excellent quality (13–16). This approach allowed for a standardized quality assessment across the heterogeneous studies included in our analysis [16].

### Statistical analysis

Statistical analyses were conducted using R studio (version 2024.09.0 + 375) with the “meta” and “metafor” packages. For dichotomous outcomes, we calculated proportions with 95% confidence intervals (CIs). Heterogeneity among studies was assessed using Cochrane’s Q test and quantified using the I² statistic, with I² values of 25%, 50%, and 75% representing low, moderate, and high heterogeneity, respectively [8].

Given the anticipated heterogeneity between studies, we employed random-effects models using the DerSimonian and Laird method for all analyses [5]. For studies reporting zero events, we applied a continuity correction by adding 0.5 to both the event and non-event counts to enable proportion calculation. We performed separate meta-analyses for precranioplasty hydrocephalus rates, postcranioplasty hydrocephalus rates, shunt-dependent hydrocephalus rates, and resolution of hydrocephalus after cranioplasty.

Sensitivity analyses were conducted using leave-one-out methodology to evaluate the influence of individual studies on pooled estimates and heterogeneity. Meta-regression analyses were performed to explore potential sources of heterogeneity, including the publication year and timing of cranioplasty after decompressive craniectomy. We directly compared the pre- and post-cranioplasty hydrocephalus rates using meta-regression to test for statistically significant differences.

Publication bias was assessed visually using funnel plots and statistically using Egger’s regression test [6]. Statistical significance was set at *p* < 0.05 for all analyses.

## Results

### Study selection

**Fig. 1:**
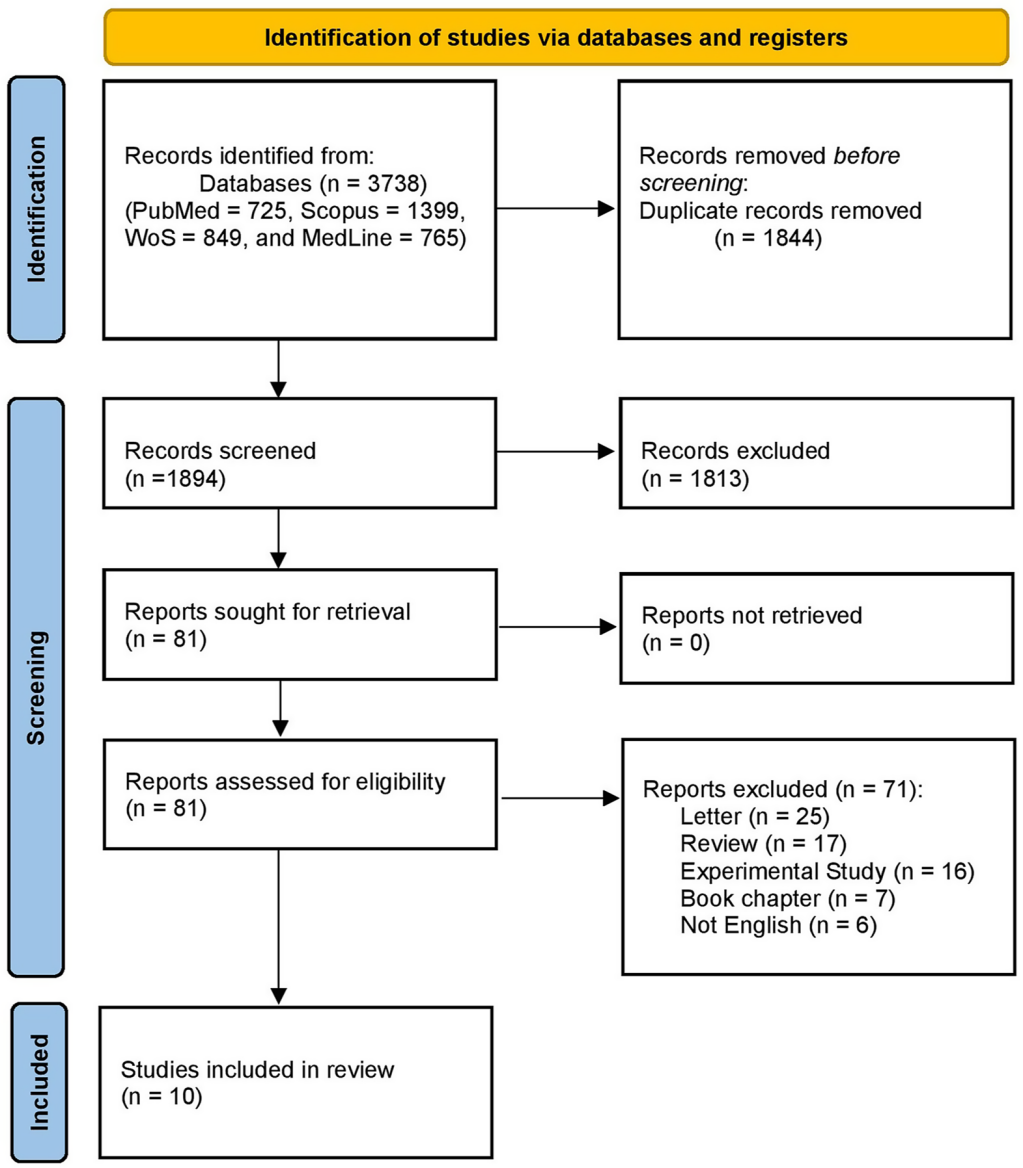
PRISMA flow diagram illustrating the study selection process, detailing the identification, screening, eligibility assessment, and final inclusion of studies in the meta-analysis

We searched the databases for 3738 records. After removing 1844 duplicates, 1894 references remained for primary screening by title and abstract. Following this initial screening, 1813 studies were excluded based on predefined criteria, leaving 81 articles for full-text assessment. After a detailed evaluation of the complete texts, 71 studies were excluded because of insufficient data, inappropriate study design, or irrelevant outcomes. This resulted in ten studies [10, 15, 20–27]. met al.l inclusion criteria. The PRISMA flowchart of the selection process is shown in Fig. 1.

### Study characteristics

#### Hydrocephalus following decompressive craniectomy for ischemic stroke

Ten studies examining hydrocephalus following decompressive craniectomy (DC) for ischemic stroke were included in this meta-analysis. The studies were conducted across seven countries: Germany (2 studies), Taiwan (2 studies), Japan (2 studies), the USA (2 studies), China (1 study), and Canada (1 study). All studies had a retrospective design, with publication dates ranging from 2007 to 2019.

The total sample size across all studies was 579 patients who underwent decompressive craniectomy, with individual study populations ranging from 13 to 198 patients. The mean age of the patients ranged from 44 to 63.5 years, with most studies reporting a predominance of male patients (38–82%). The study period spanned from 1998 to 2016, with follow-up durations varying from one month to two years, where specified.

All studies focused on malignant middle cerebral artery (MCA) infarction, with some including internal carotid artery (ICA) infarction. The primary indication for DC across studies was medical refractory, increased intracranial pressure, or deteriorating consciousness with a significant mass effect. The timing of DC ranged from 0 to 5 days after stroke onset, with most procedures performed within the first 48 h.

Definitions of hydrocephalus varied between studies, with most studies using radiographic criteria such as Evans index > 0.3, ventricular dilatation on CT, or clinical improvement after CSF drainage. Exclusion criteria also differed between studies, but commonly included early mortality, intracranial infection, subarachnoid hemorrhage, or intraventricular hemorrhage. More detailed information on the patient characteristics and study specifics can be found in Table 1.

**Table 1:** Baseline characteristics of studies on hydrocephalus following decompressive craniectomy for ischemic stroke

Study	Country	Study Design	Sample Size	Mean Age (years)	Male (%)	Study Period	Type of Stroke	DC Indication	Timing of DC (days from onset)	Definition of Hydrocephalus	Exclusion Criteria	Duration of Follow-up
Schwake et al. 2019	Germany	Retrospective	198 (12 with strokectomy)	57.3 ± 12.0	62.6%	2009–2016	Malignant MCA/ICA infarction	Medically refractory ICP	Median 1 day (range 0–2)	Ventricular dilatation requiring CSF diversion	None specified	Not specified
Finger et al. 2017	Germany	Retrospective	99	52.9 ± 11.7	62%	2005–2013	Malignant MCA infarction	Space-occupying MCA infarction	Not specified	Clinical improvement after CSF drainage (RASS score)	None specified	Not specified
Lee et al. 2012	Taiwan	Retrospective	17	63.2 ± 12.5	82%	2003–2006	Malignant MCA infarction	Medically refractory IICP	2.58 ± 0.71 days	Ventricular dilation on serial brain CT	Epidural abscess, death within 10 days, lost to follow-up	1 year
Nalbach et al. 2012	USA	Retrospective	39 (34 survived > 3 days)	Not specified	Not specified	2007–2009	MCA infarction	Medically refractory ICP	Not specified	Extra-axial fluid collections > 5 mm exerting mass effect	Death on or prior to POD 3	Until cranioplasty
Lin et al. 2015	Taiwan	Retrospective	13	61.8 ± 15.0	38%	2005–2011	Malignant MCA/ICA infarction	Consciousness deterioration or ICP elevation	1.84 ± 0.84 days	Modified Evans index > 0.3	Previous VP shunt, bilateral DC, acute hydrocephalus at admission, death within 1 month	Not specified
Takeuchi et al. 2013	Japan	Retrospective	28	63.5 ± 10.1	50%	15-year period	Malignant MCA/ICA infarction	Deteriorating consciousness, significant mass effect	64.3% within 48 h	Evans index > 0.3 with narrowed CSF spaces	Death or lost within 1 month	Not specified
Takeuchi et al. 2013 (Acta)	Japan	Retrospective	23	60.8	57%	Not specified	Malignant MCA/ICA infarction	Deteriorating consciousness, significant mass effect	2.4 days (mean)	Evans index > 0.3 with narrowed CSF spaces	Not specified	173 days (mean)
Wang et al. 2015	China	Retrospective	128	52.6 ± 11.0	52.3%	2004–2014	Malignant hemispheric cerebral infarction	Medically refractory ICP	Not specified	Evans index > 0.3 with narrowed CSF spaces	Intracranial infection, massive hemorrhagic transformation, survival < 6 months	726 days (mean)
Rahme et al. 2010	Canada	Retrospective	17	Median 44 (range 27–53)	47%	2001–2009	Malignant MCA infarction, hemorrhagic CVA, dural sinus thrombosis	Deteriorating consciousness, significant mass effect	Not specified	Clinical deterioration with radiographic evidence	SAH, IVH, head trauma	24 months (median)
Waziri et al. 2007	USA	Retrospective	17	47.8	53%	1998–2005	Hemispheric cerebral infarction, deep ICH	Medically refractory ICP	Within 5 days	Radiographic ventricular dilatation or extra-axial CSF	SAH, IVH, intracranial infection	Not specified

**Abbreviations**: DC = Decompressive Craniectomy; MCA = Middle Cerebral Artery; ICA = Internal Carotid Artery; ICP = Intracranial Pressure; IICP = Increased Intracranial Pressure; CT = Computed Tomography; CSF = Cerebrospinal Fluid; SAH = Subarachnoid Hemorrhage; IVH = Intraventricular Hemorrhage; ICH = Intracerebral Hemorrhage; POD = Post-Operative Day; RASS = Richmond Agitation Sedation Scale; VP = Ventriculoperitoneal

### Risk of bias

Based on the cumulative scores, studies were categorized as: extremely poor quality (0–4), low quality (5–7), fair quality (8–12), and excellent quality (13–16). This approach allowed for a standardized quality assessment across the heterogeneous studies included in our analysis. As shown in Table 2, the majority of included studies (6/10) were of fair quality, with scores ranging from to 9–11 points. Three studies were classified as low-quality (6–7 points), and one study was considered extremely poor-quality (4 points). The most common methodological limitations were the lack of prospective data collection (all studies scored 0), absence of prospective study size calculation (all studies scored 0), and inadequate follow-up reporting (7/10 studies scored 1 or 0) (Table 2).

**Table 2:** Assessment of the quality of studies through methodological index for Non-Randomized studies (MINORS).

Study ID	Clearly Stated Aim	Consecutive Patients	Prospective Collection Data	Endpoints Appropriate	Unbiased Assessment of Endpoints	Follow-up Period Appropriate	Loss to Follow-up < 5%	Prospective Study Size Calculation	MINORS Score
Schwake et al. 2019	2	2	0	2	2	1	1	0	10/16
Finger et al. 2017	2	2	0	2	1	1	1	0	9/16
Lee et al. 2012	2	1	0	2	1	2	1	0	9/16
Nalbach et al. 2012	1	2	0	1	1	1	1	0	7/16
Lin et al. 2015	2	1	0	2	2	1	1	0	9/16
Takeuchi et al. 2013	2	1	0	2	2	1	1	0	9/16
Takeuchi et al. 2013 (Acta)	2	1	0	2	2	2	1	0	10/16
Wang et al. 2015	2	2	0	2	2	2	1	0	11/16
Rahme et al. 2010	1	1	0	1	1	2	1	0	7/16
Waziri et al. 2007	1	1	0	1	1	1	1	0	6/16

*MINORS scoring:* 0 = not reported; 1 = reported but inadequate; 2 = reported and adequate; Maximum score for non-comparative studies = 16

### Quality of evidence

Based on the GRADE assessment of evidence quality for hydrocephalus outcomes following decompressive craniectomy for ischemic stroke, our meta-analysis revealed significant limitations in the available evidence. Most outcomes, including pre- and post-cranioplasty hydrocephalus rates and ventriculomegaly rates, were supported by very low-quality evidence (⊕◯◯◯), primarily due to the retrospective design of all included studies, very high statistical heterogeneity (I² > 89%), and suspected publication bias. More favorable evidence quality was observed for shunt-dependent hydrocephalus and resolution of hydrocephalus after cranioplasty, both assessed as low-quality evidence (⊕⊕◯◯) due to more moderate heterogeneity (I² = 61.4% and 68.7%, respectively). Despite the high clinical importance of all outcomes examined, the predominance of fair-quality studies (6/10) with methodological limitations, including lack of prospective data collection, absence of study size calculation, and inadequate follow-up reporting, significantly undermine the strength of recommendations that can be made. Future research employing prospective designs with standardized outcome definitions and adequate follow-up periods is essential to improve the quality of evidence and guide clinical decision-making in this area (Table 3).

### Hydrocephalus and ventriculomegaly rates in patients with cranioplasty: meta-analysis results

#### Pre-cranioplasty hydrocephalus rate

**Fig. 2:**
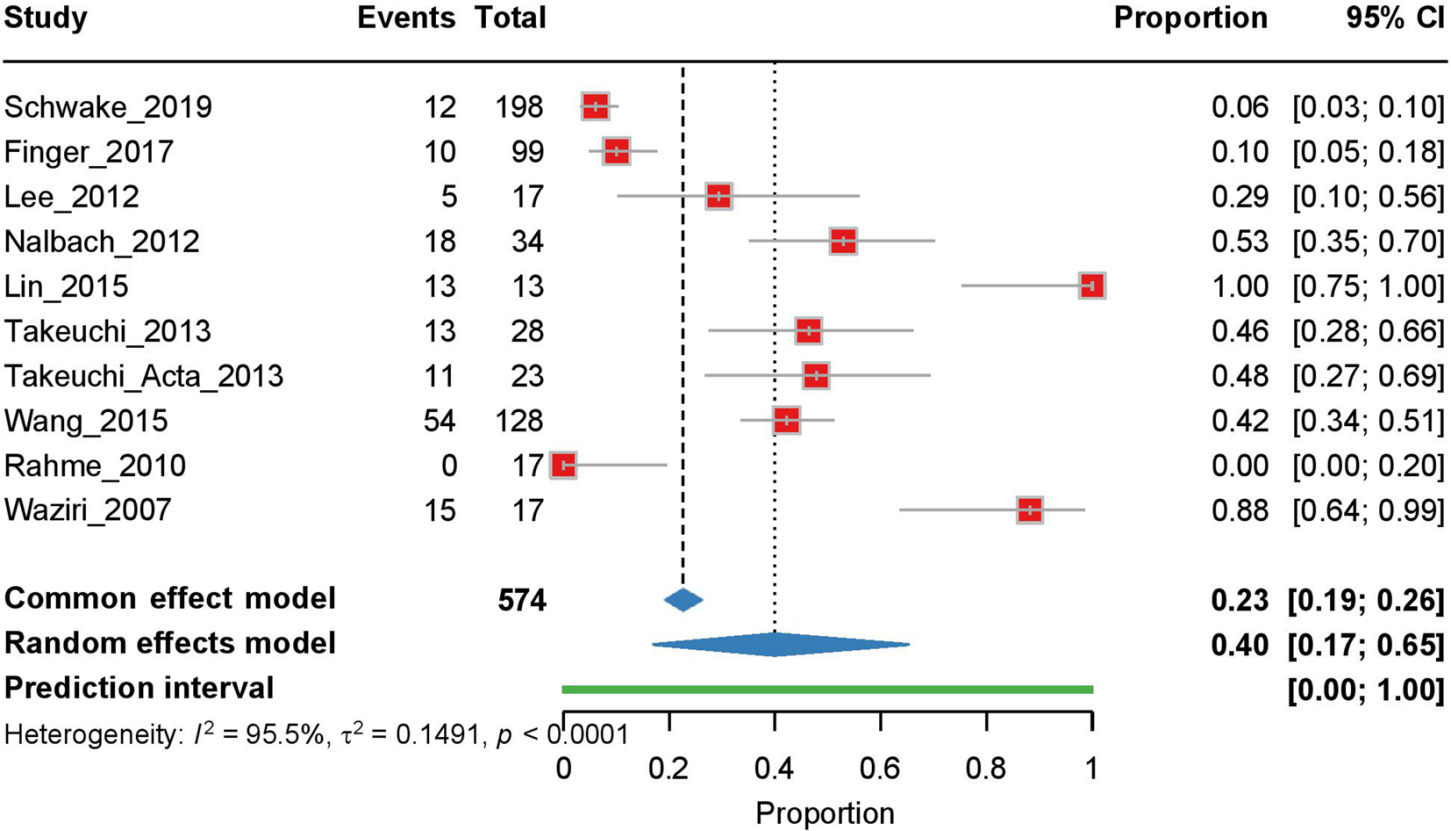
Forest plot of the meta-analysis of pre-cranioplasty hydrocephalus rates. Individual study proportions with 95% confidence intervals (CI) are shown, along with the pooled proportion estimate calculated using a random-effects model. The prediction interval (PI) is also indicated

Our meta-analysis included 10 studies that examined the rate of pre-cranioplasty hydrocephalus. Owing to significant heterogeneity between studies (I² = 95.5%, τ² = 0.1491, *p* < 0.0001), we applied a random-effects model. The pooled estimate showed a pre-cranioplasty hydrocephalus rate of 0.40 [95% CI: 0.17–0.65], with individual study rates ranging from 0.00 (Rahme et al., 2010) to 1.00 (Lin et al., 2015) (Fig. 2). The sensitivity analysis revealed that removing Lin et al. (2015) had the most substantial impact on the pooled estimate, reducing it to 0.337, indicating the influence of this study on the overall results (Supplementary Fig. 1).

#### Post-cranioplasty hydrocephalus rate

**Fig. 3:**
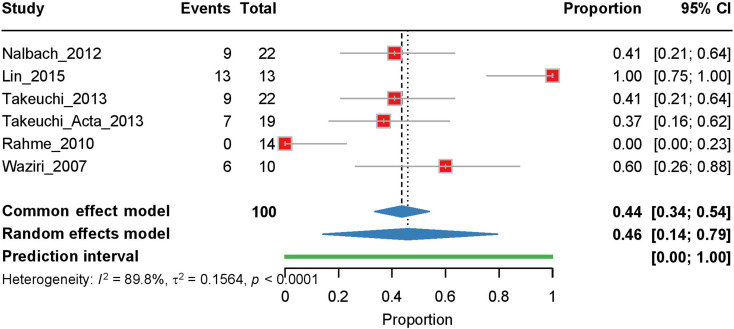
Forest plot of the meta-analysis of post-cranioplasty hydrocephalus rates. Individual study proportions with 95% CI are shown, along with the pooled proportion estimate calculated using a random-effects model. The PI is also indicated

Six studies were included in our analysis of postcranioplasty hydrocephalus. The random effects model, employed due to high heterogeneity (I² = 89.8%, τ² = 0.1564, *p* < 0.0001), yielded a pooled estimate of 0.46 [95% CI: 0.14–0.79], with a prediction interval of 0.00–1.00 (Fig. 3). Similar to the pre-cranioplasty analysis, Lin et al. (2015) showed the greatest influence on the results, with their exclusion reducing the estimate to 0.352 (Supplementary Fig. 2).

#### Pre-cranioplasty ventriculomegaly rate

**Fig. 4:**
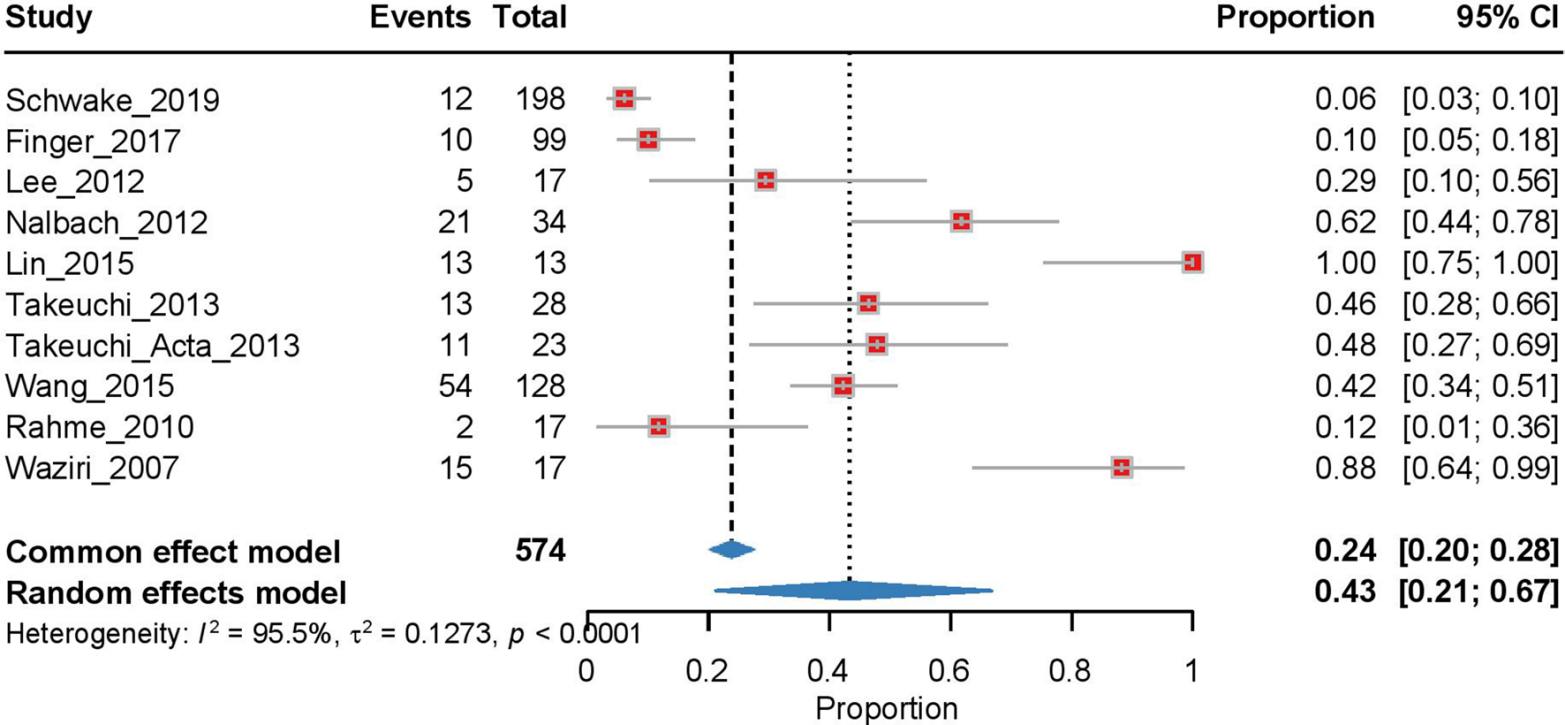
Forest plot of the meta-analysis of pre-cranioplasty ventriculomegaly rates. Individual study proportions with 95% CI are shown, along with the pooled proportion estimate calculated using a random-effects model. The PI is also indicated

Our meta-analysis of 10 studies examining pre-cranioplasty ventriculomegaly demonstrated significant heterogeneity (I² = 95.5%, τ² = 0.1273, *p* < 0.0001). Using a random effects model, we found a pooled ventriculomegaly rate of 0.43 [95% CI: 0.21–0.67], with individual study rates showing substantial variation from 0.06 (Schwake et al., 2019) to 1.00 (Lin et al., 2015; Fig. 4). The sensitivity analysis identified Lin et al. (2015) as having the greatest impact on the pooled estimate, with its removal reducing the estimate to 0.361 (Supplementary Fig. 3).

#### Post-cranioplasty ventriculomegaly rate

**Fig. 5:**
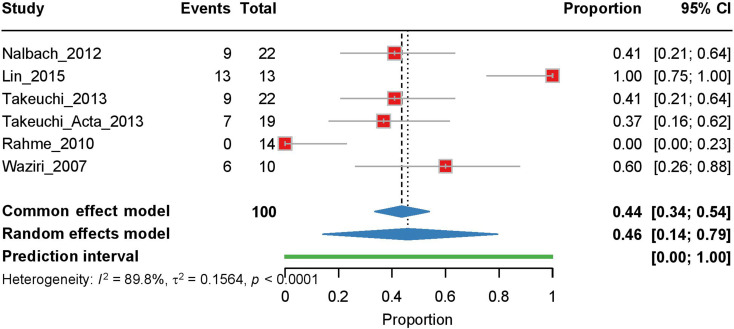
Forest plot of the meta-analysis of post-cranioplasty ventriculomegaly rates. Individual study proportions with 95% CI are shown, along with the pooled proportion estimate calculated using a random-effects model. The PI is also indicated

Six studies that assessed post-cranioplasty ventriculomegaly were included in our analysis. Given the substantial heterogeneity (I² = 89.8%, τ² = 0.1564, *p* < 0.0001), we employed a random effects model, which yielded a pooled estimate of 0.46 [95% CI: 0.14–0.79], with a prediction interval of 0.00–1.00 (Fig. 5). Consistent with other outcomes, the removal of Lin et al. (2015) from the sensitivity analysis had the most pronounced effect, decreasing the estimate to 0.352 (Supplementary Fig. 4).

#### Shunt-dependent hydrocephalus rate

**Fig. 6:**
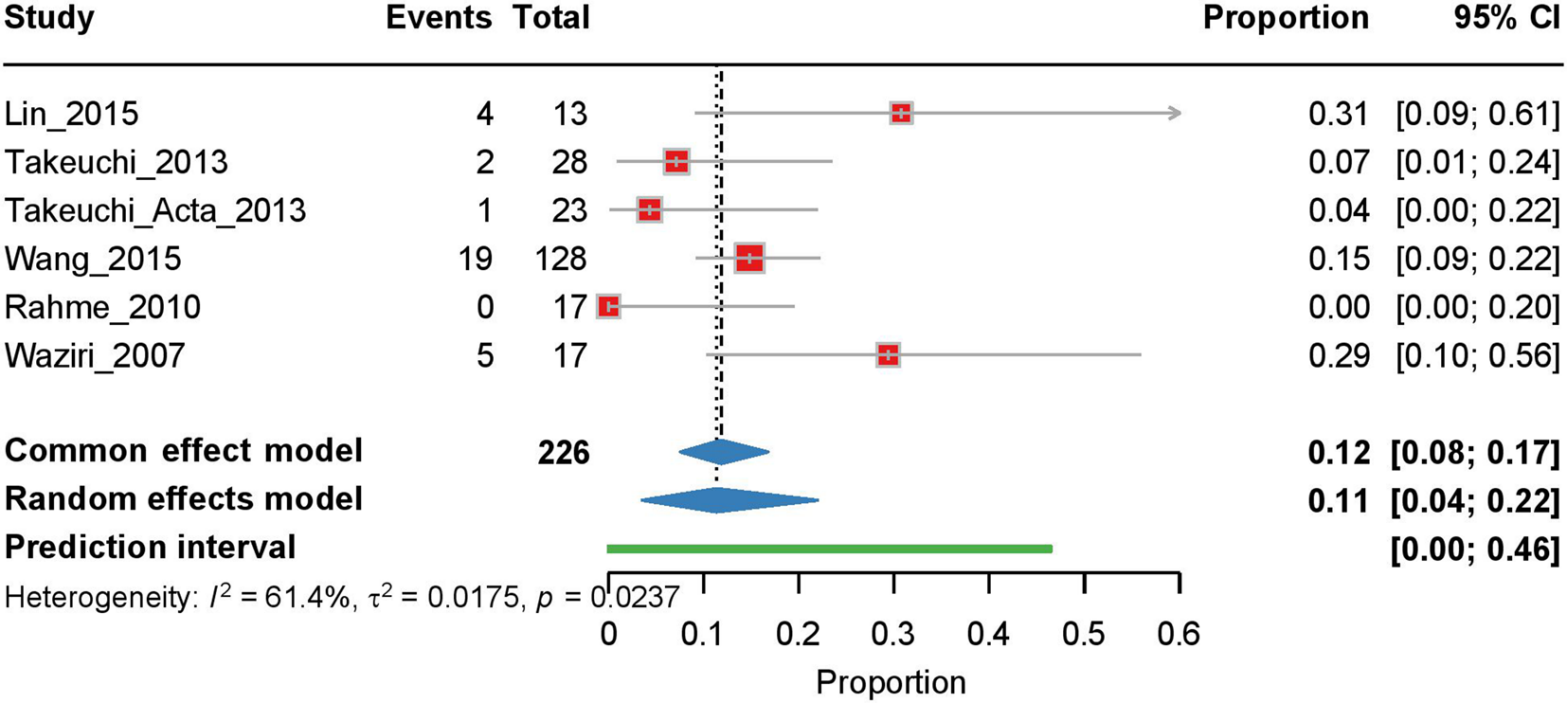
Forest plot of the meta-analysis of shunt-dependent hydrocephalus rates. Individual study proportions with 95% CI are shown, along with the pooled proportion estimate calculated using a random-effects model. The PI is also indicated

Our analysis of six studies on shunt-dependent hydrocephalus showed moderate heterogeneity (I² = 61.4%, τ² = 0.0175, *p* = 0.0237). The random effects model yielded a rate of 0.11 [95% CI: 0.04–0.22] with a prediction interval of 0.00-0.46 (Fig. 6). Meta-regression analysis by publication year revealed no significant temporal trend (*p* = 0.804, R² = 0.015, Pearson’s *r* = -0.121) (Supplementary Fig. 5).

#### Resolution of hydrocephalus after cranioplasty

**Fig. 7:**
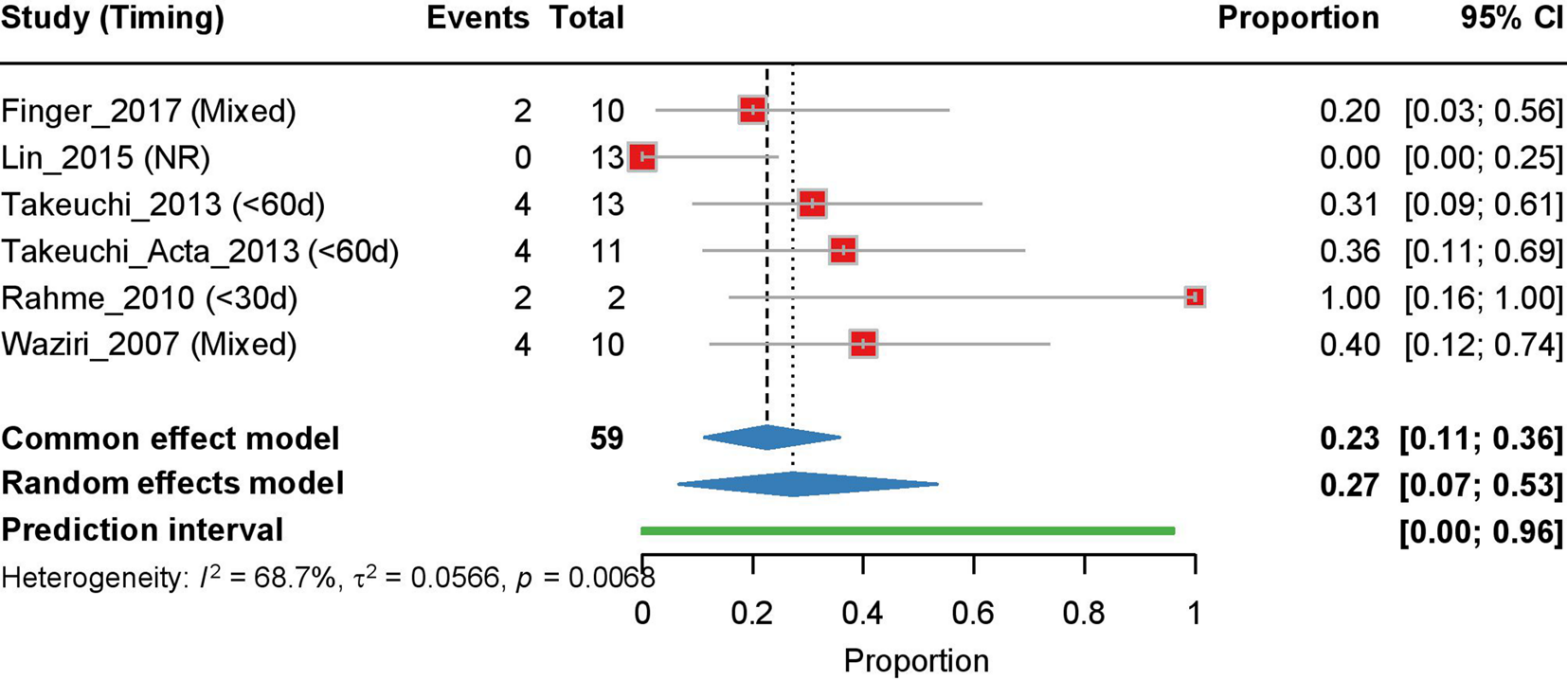
Forest plot of the meta-analysis of hydrocephalus resolution rates after cranioplasty. Individual study proportions with 95% CI are shown, along with the pooled proportion estimate calculated using a random-effects model. The PI is also indicated

Six studies that examined hydrocephalus resolution after cranioplasty were included. With moderate heterogeneity (I² = 68.7%, τ² = 0.0566, *p* = 0.0068), the random effects model provided an estimate of 0.27 [95% CI: 0.07–0.53] with a prediction interval of 0.00-0.96 (Fig. 7). Meta-regression analysis investigating the relationship between cranioplasty timing and hydrocephalus resolution showed a negative correlation (*r* = -0.788), suggesting that earlier cranioplasty might be associated with higher resolution rates, although this was not statistically significant (*p* = 0.255, R² = 0.621) (Supplementary Fig. 6).

#### Comparison between pre and post-cranioplasty hydrocephalus

**Fig. 8:**
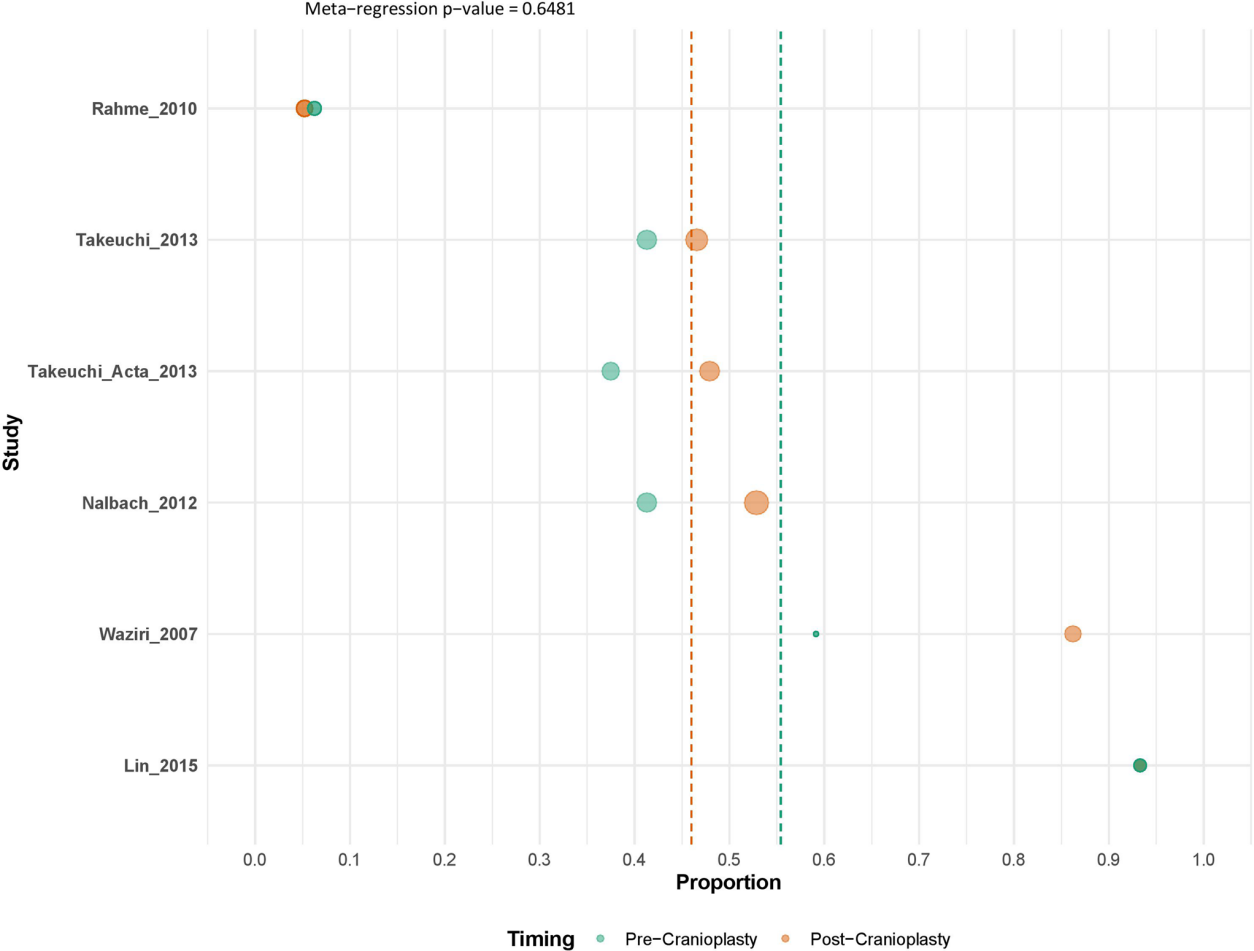
Meta-regression plot comparing pre- and post-cranioplasty hydrocephalus rates. The plot visualizes the relationship between the two time points across included studies, with the regression line indicating the overall trend and associated p-value for the comparison

A direct comparison between pre- and post-cranioplasty hydrocephalus rates revealed no statistically significant difference (meta-regression *p* = 0.6481) (Fig. 8). Box plot visualization demonstrated slightly higher post-cranioplasty rates, but with substantial overlap between the distributions, suggesting minimal clinical difference between the two timepoints (Supplementary Fig. 7).

#### Sensitivity analyses and heterogeneity

Sensitivity analyses identified several influential studies across the outcomes. For shunt-dependent hydrocephalus, excluding Waziri et al. (2007), heterogeneity was substantially reduced from I² = 49.1–29.8% (Supplementary Fig. 8). This study was a potential source of between-study variation. Similar influential patterns were observed for other outcomes, highlighting the need for a cautious interpretation of the pooled results.

### Publication bias

Funnel plot asymmetry was observed across all hydrocephalus outcomes (Supplementary Fig. 9), with several studies falling outside the expected boundaries, suggesting a potential publication bias. This observation underscores the importance of considering unpublished data and the possibility that the available literature does not represent the full spectrum of clinical findings.

## Discussion

Decompressive craniectomy (DC) for malignant ischemic stroke is a lifesaving procedure; however, hydrocephalus is a possible complication and can have long-term effects on outcomes [10, 12, 17]. Although hydrocephalus after DC has been established, especially in the context of TBI [2, 3, 9, 13], its specific postoperative course regarding CP in stroke patients is not well described. This meta-analysis was performed to determine the rates of hydrocephalus and ventriculomegaly before and after CP, the need for permanent CSF diversion, and investigate hydrocephalus resolution following CP in patients who underwent DC for ischemic stroke.

Our pooled analysis revealed substantial rates of radiographically defined hydrocephalus (40%) and ventriculomegaly (43%) already present before cranioplasty in this patient population. Following cranioplasty, these rates remained similarly high (46% for both outcomes), with our direct comparison showing no statistically significant difference between the pre- and post-CP time points (*p* = 0.6481). This finding suggests that at the pooled level within the timeframe assessed by these studies, cranioplasty itself does not consistently lead to an immediate resolution or worsening of ventricular enlargement established after DC. The underlying CSF dynamic disruption occurring after the initial large DC may be the primary driver, often persisting throughout the CP period.

Importantly, regardless of the high rate of radiographic hydrocephalus or ventriculomegaly (greater than 40%), the rate of shunt-dependent hydrocephalus was estimated to be 11% [95% CI: 0.04–0.22]. The clinical difference (requiring treatment) is essential to know and this article highlights that ventricular dilation is not clinically equivalent to hydrocephalus or an indication for surgical treatment even in the absence of ventricular catheter (for instance), since ventricular expansion in the standard range can also be seen in healthy patients. In this context, detailed clinical and neurological assessments with neuroimaging are essential when considering CSF diversion, as many patients with ventriculomegaly may not benefit from shunting.

Furthermore, our analysis indicated that approximately 27% [95% CI: 0.07–0.53] of patients experienced resolution of hydrocephalus after cranioplasty. While our meta-regression exploring the impact of CP timing did not reach statistical significance (*p* = 0.255), the observed negative correlation (*r* = -0.788) suggests that earlier cranioplasty might contribute to hydrocephalus resolution. This aligns with the hypothesis that restoring cranial integrity earlier could help normalize CSF dynamics and brain compliance [12]. However, given the non-significant result and limitations discussed below, this remains speculative and requires a dedicated prospective investigation.

Our findings partially resonate with the meta-analysis by Ovenden et al. [14], who reported a pooled hydrocephalus/ventriculomegaly rate of 38% and shunt rate of 10% following DC for ischemic stroke (including studies with and without subsequent CP). Our slightly higher hydrocephalus rates (40–46%) and similar shunt dependency rates (11%) fall within comparable ranges. However, our study distinctively focused on the cranioplasty window, allowing for pre- vs. post-CP comparison and analysis of resolution, providing specific insights into the dynamics around this secondary procedure; for example, Ovenden et al. did not analyze them separately.

### Strengths and limitations

A key strength of our analysis is the specific focus on the cranioplasty timeframe, which enabled a direct comparison of hydrocephalus rates before and after skull reconstruction. However, several significant limitations of this study should be considered when interpreting our findings. First, the exclusive reliance on retrospective studies introduces inherent risks of selection bias, information bias, and confounding. The “fair” to “extremely poor” quality ratings based on MINORS criteria [16], primarily due to lack of prospective data collection and inadequate follow-up, further weaken the evidence base. This is reflected in the overall GRADE assessment, indicating very low-to low-quality evidence for most outcomes (Table 3).

**Table 3:** GRADE SUMMARY: hydrocephalus following decompressive craniectomy for ischemic stroke

Outcome	No. of Studies	Study Design	Risk of Bias	Inconsistency	Indirectness	Imprecision	Other Considerations	Effect (95% CI)	Certainty	Importance
Pre-Cranioplasty Hydrocephalus Rate	10	Retrospective Cohort	Moderate (6 studies: fair quality, 3 studies: low quality, 1 study: extremely poor quality)	Very High (I² = 95.5%)	None	None	Publication bias suspected	0.40 (0.17–0.65)	Very Low (⊕◯◯◯)	High (★★★★☆)
Post-Cranioplasty Hydrocephalus Rate	6	Retrospective Cohort	Moderate (majority fair quality)	Very High (I² = 89.8%)	None	None	Publication bias suspected	0.46 (0.14–0.79)	Very Low (⊕◯◯◯)	High (★★★★☆)
Pre-Cranioplasty Ventriculomegaly Rate	10	Retrospective Cohort	Moderate (6 studies: fair quality, 3 studies: low quality, 1 study: extremely poor quality)	Very High (I² = 95.5%)	None	None	Publication bias suspected	0.43 (0.21–0.67)	Very Low (⊕◯◯◯)	High (★★★★☆)
Post-Cranioplasty Ventriculomegaly Rate	6	Retrospective Cohort	Moderate (majority fair quality)	Very High (I² = 89.8%)	None	None	Publication bias suspected	0.46 (0.14–0.79)	Very Low (⊕◯◯◯)	High (★★★★☆)
Shunt-Dependent Hydrocephalus Rate	6	Retrospective Cohort	Moderate (majority fair quality)	Moderate (I² = 61.4%)	None	None	Publication bias suspected	0.11 (0.04–0.22)	Low (⊕⊕◯◯)	High (★★★★☆)
Resolution of Hydrocephalus After Cranioplasty	6	Retrospective Cohort	Moderate (majority fair quality)	Moderate (I² = 68.7%)	None	None	Publication bias suspected	0.27 (0.07–0.53)	Low (⊕⊕◯◯)	High (★★★★☆)

*Quality Assessment Criteria*: Extremely poor quality (0–4), Low quality (5–7), Fair quality (8–12), and Excellent quality (13–16)

*GRADE Certainty Ratings*: High (⊕⊕⊕⊕), Moderate (⊕⊕⊕◯), Low (⊕⊕◯◯), Very Low (⊕◯◯◯)

*Importance*: Critical (★★★★★), High (★★★★☆), Moderate (★★★☆☆), Low (★★☆☆☆), Not Important (★☆☆☆☆)

Second, the substantial statistical heterogeneity (I² > 89% for most hydrocephalus/ventriculomegaly outcomes) across studies is a major concern, suggesting significant variability in patient populations, hydrocephalus definitions, imaging protocols, timing of assessments, and potential surgical techniques or perioperative management. This variability limits the reliability of the pooled estimates and underscores the wide prediction intervals, indicating considerable uncertainty regarding the true effect in individual settings. While sensitivity analyses identified influential studies (e.g., Lin et al. [12]), heterogeneity remained high for most outcomes.

Third, inconsistent definitions of hydrocephalus and ventriculomegaly across studies likely contributed to the heterogeneity and made direct comparisons challenging. Furthermore, variability in the follow-up duration may lead to under-ascertainment of delayed hydrocephalus or shunt requirements. Finally, the observed funnel plot asymmetry suggests potential publication bias, where studies with less “significant” or negative findings might be underrepresented. The lack of randomized controlled data, particularly comparing different CP timings or management strategies, prevents definitive conclusions regarding causality.

#### Future directions

However, further research is required to address these limitations. Large multicenter prospective cohort studies or ideally randomized controlled trials (RCTs) are needed. These studies should employ standardized, consensus-based definitions for hydrocephalus and ventriculomegaly, predefined imaging protocols and assessment time points (relative to both DC and CP), and sufficiently long follow-up periods to capture delayed events and functional outcomes. Investigating specific predictors (e.g., initial stroke severity, DC size, presence of hygroma [13], and timing of CP) for developing shunt-dependent hydrocephalus versus achieving post-CP resolution would be highly valuable. Comparing specific CP timing strategies (e.g., early vs. late) within an RCT framework could clarify their impact on CSF dynamics and neurological recovery.

## Conclusion

In summary, this meta-analysis demonstrated that HC and ventriculomegaly were relatively common (40–46%) in HD and non-HD patients undergoing cranioplasty following decompressive craniectomy for ischemic stroke and that the overall pooled data did not detect a significant change in C London HC immediately post-CP. However, only a small percentage (~ 11%) required permanent CSF shunting, underscoring the importance of clinical correlation over imaging alone. Although a trend towards improved hydrocephalus resolution with earlier cranioplasty was observed, further investigation is required. The low to very low quality of evidence available is impacted by retrospective study designs and by considerable heterogeneity of previous studies, which calls for the urgent need for high-quality prospective studies to inform clinical management in this vulnerable patient population with regard to hydrocephalus.

## Electronic supplementary material

Below is the link to the electronic supplementary material.

Supplementary Material 1

Supplementary Material 2: Detailed results of sensitivity analyses (leave-one-out), meta-regression analyses exploring heterogeneity sources (publication year, cranioplasty timing), visual comparison of pre- and post-cranioplasty hydrocephalus distributions (box plot), and funnel plots assessing publication bias are provided in Supplementary Figs. 1–9.

## Data Availability

No datasets were generated or analysed during the current study.
