# Supplementary material for: Pre- and post-cranioplasty hydrocephalus in patients following decompressive craniectomy for ischemic stroke: a systematic review and meta-analysis
Source: Neurosurg Rev. 2025 Jun 18;48(1):514. doi: 10.1007/s10143-025-03650-7 (PMC12177026; doi:10.1007/s10143-025-03650-7)
Supplement: Supplementary file 1 — Supplementary Material 1 [file 10143_2025_3650_MOESM1_ESM.docx]

(Hydrocephalus OR Ventriculomegaly OR "Ventricular dilatation" OR "Ventricular dilation" OR "CSF disorder*" OR "CSF dynamic*" OR Hygroma OR "Subdural hygroma" OR "CSF leak" OR "Cerebrospinal fluid leak")

AND

("Decompressive craniectomy" OR "Decompressive hemicraniectomy" OR "Cranial decompression" OR Cranioplasty OR "Skull reconstruction" OR "Cranial reconstruction" OR "Bone flap replacement" OR "VP shunt" OR "Ventriculoperitoneal shunt" OR "CSF shunt*" OR "Cerebrospinal fluid shunt*" OR "CSF diversion" OR "Cerebrospinal fluid diversion" OR "Shunt dependent" OR "Shunt requirement")

Pubmed 725

Scopus 1,399

Wos 849

Medline 765

3738
