# Supplementary figures and images for: Pre- and post-cranioplasty hydrocephalus in patients following decompressive craniectomy for ischemic stroke: a systematic review and meta-analysis
Source: Neurosurg Rev. 2025 Jun 18;48(1):514. doi: 10.1007/s10143-025-03650-7 (PMC12177026; doi:10.1007/s10143-025-03650-7)

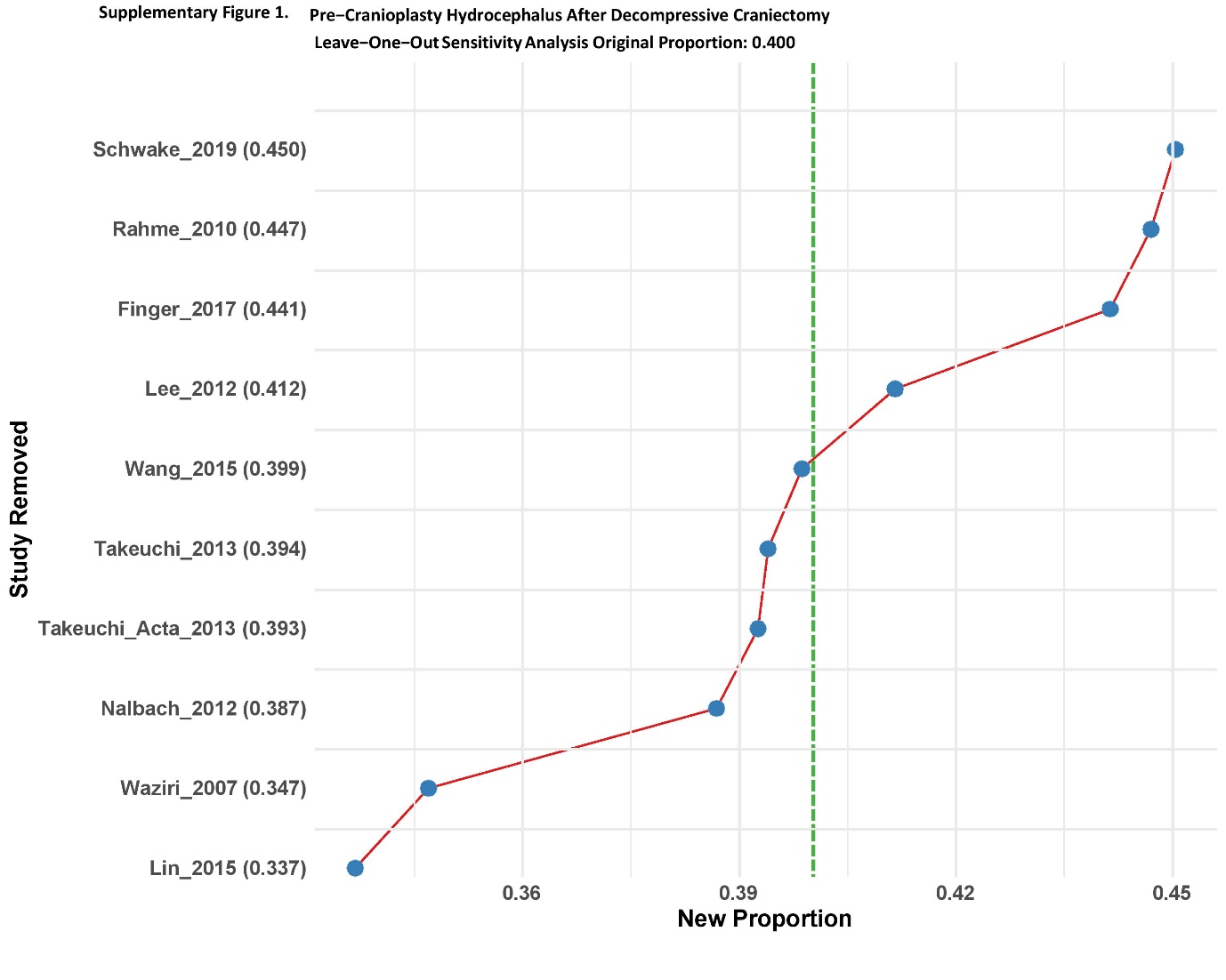


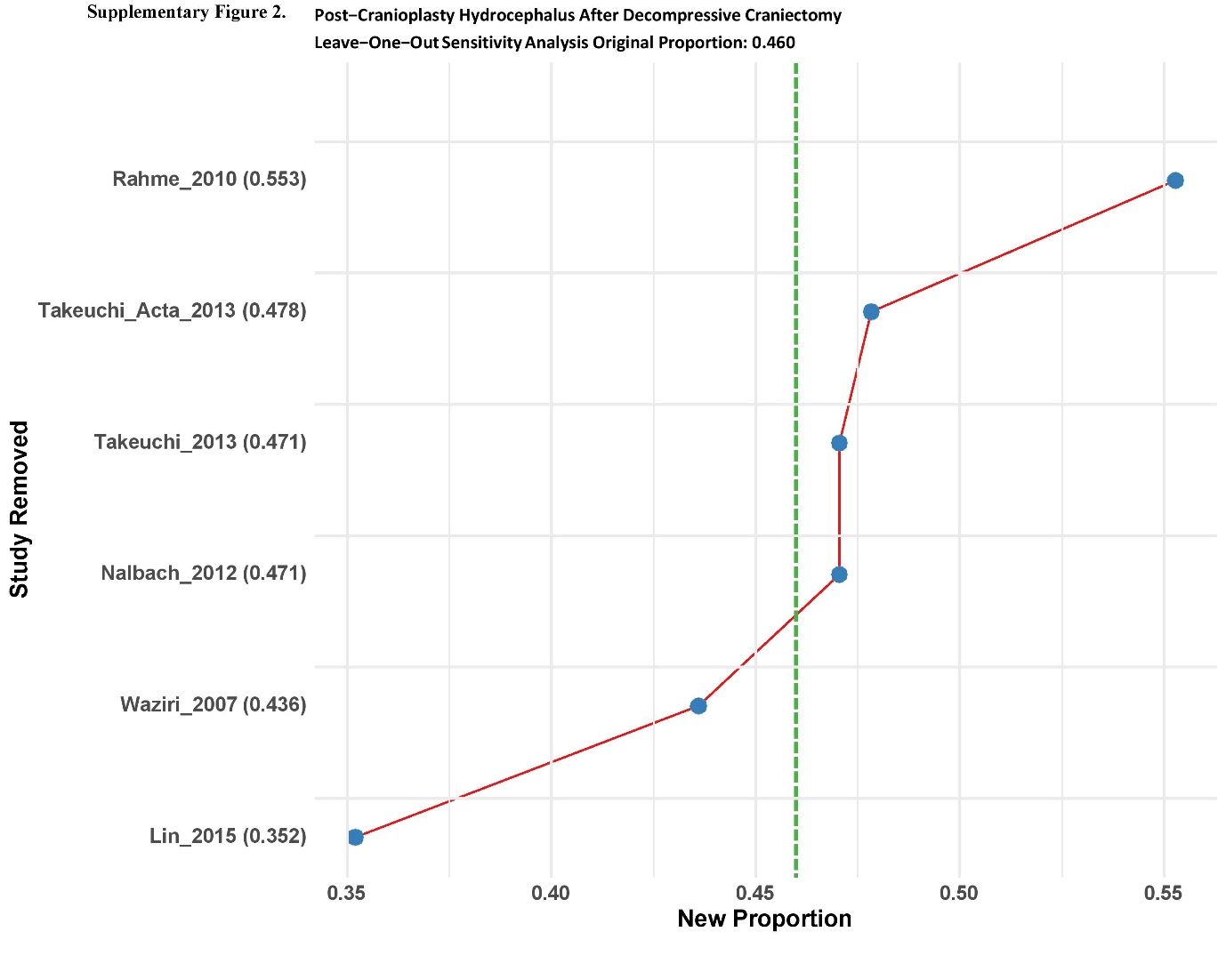


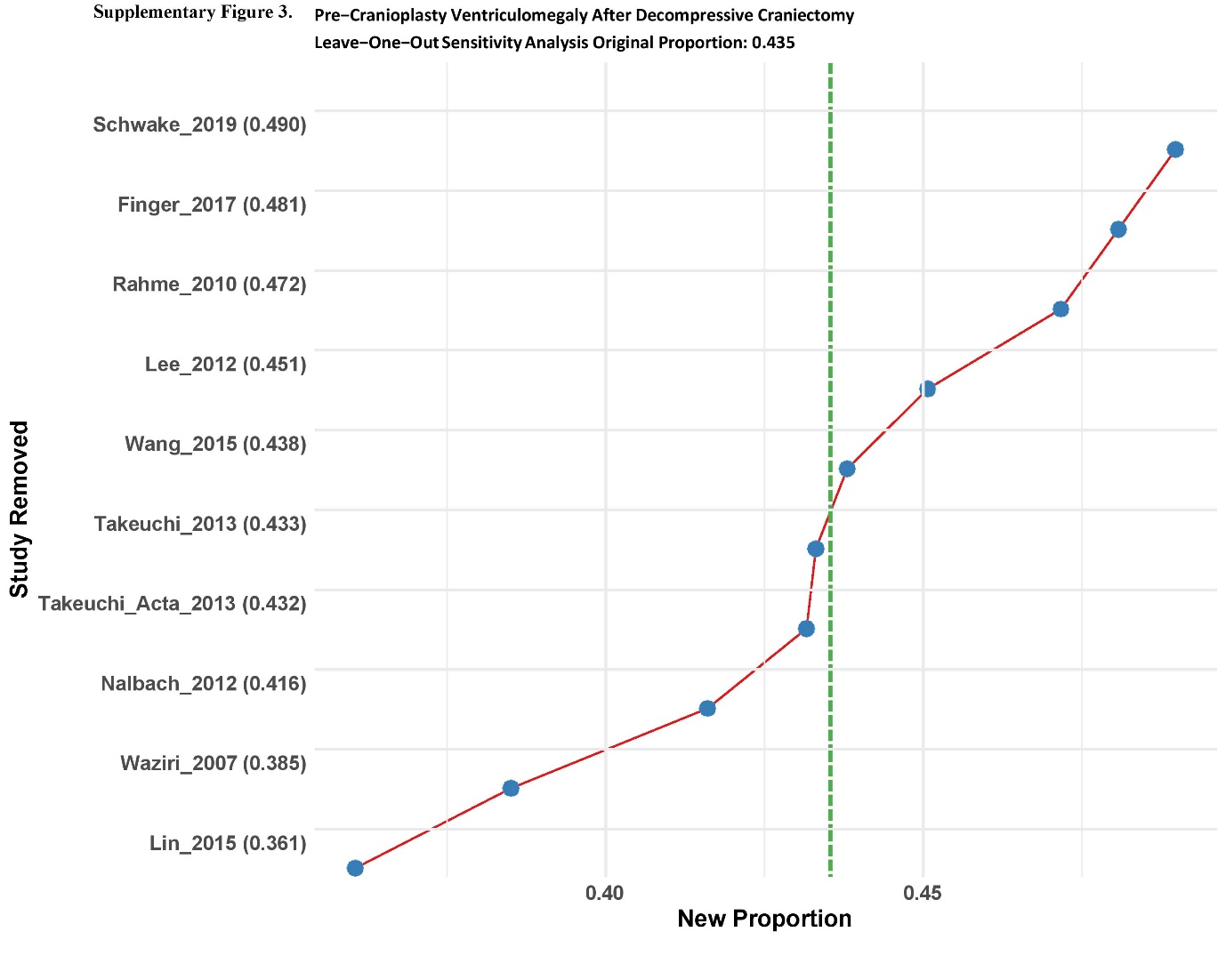


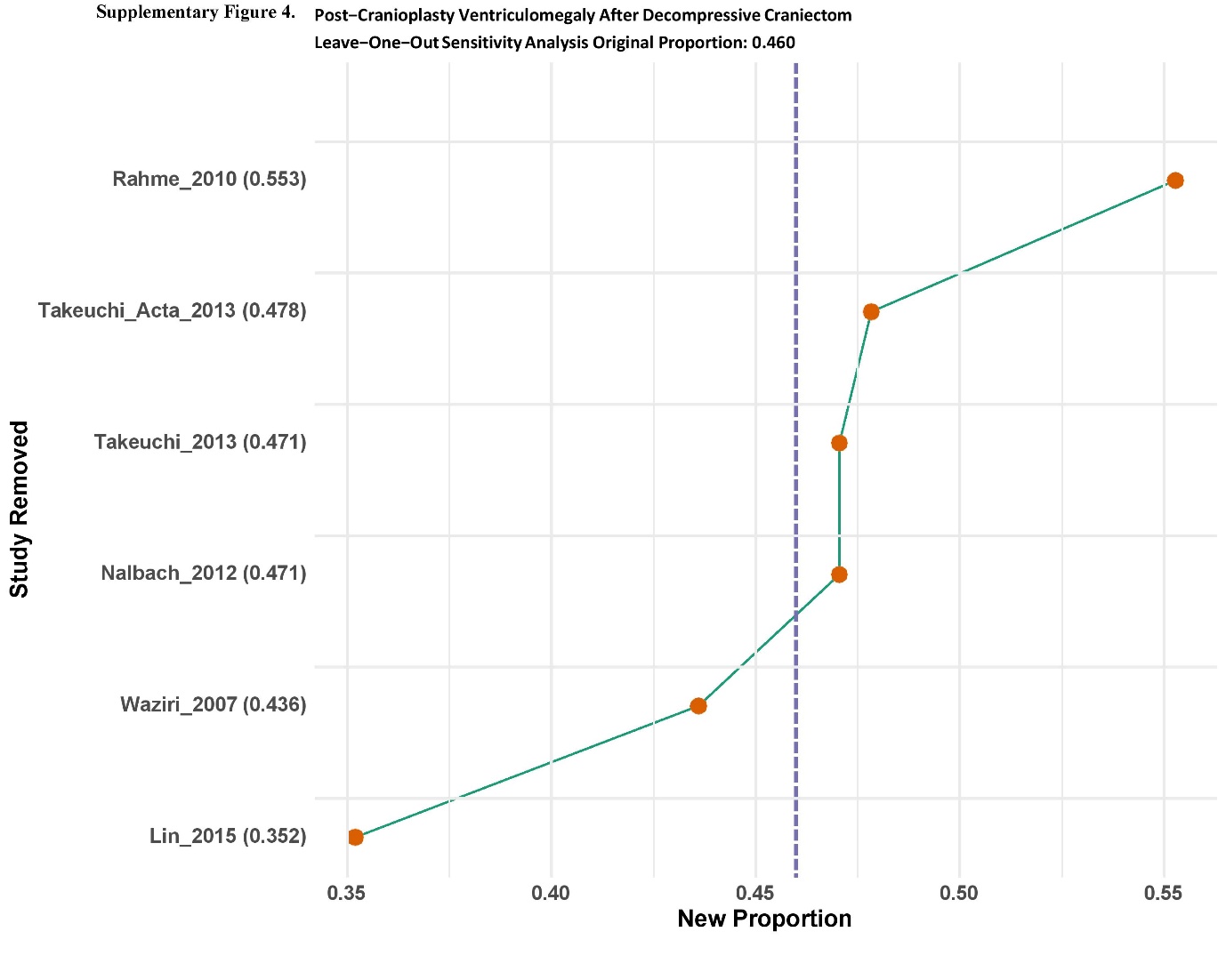


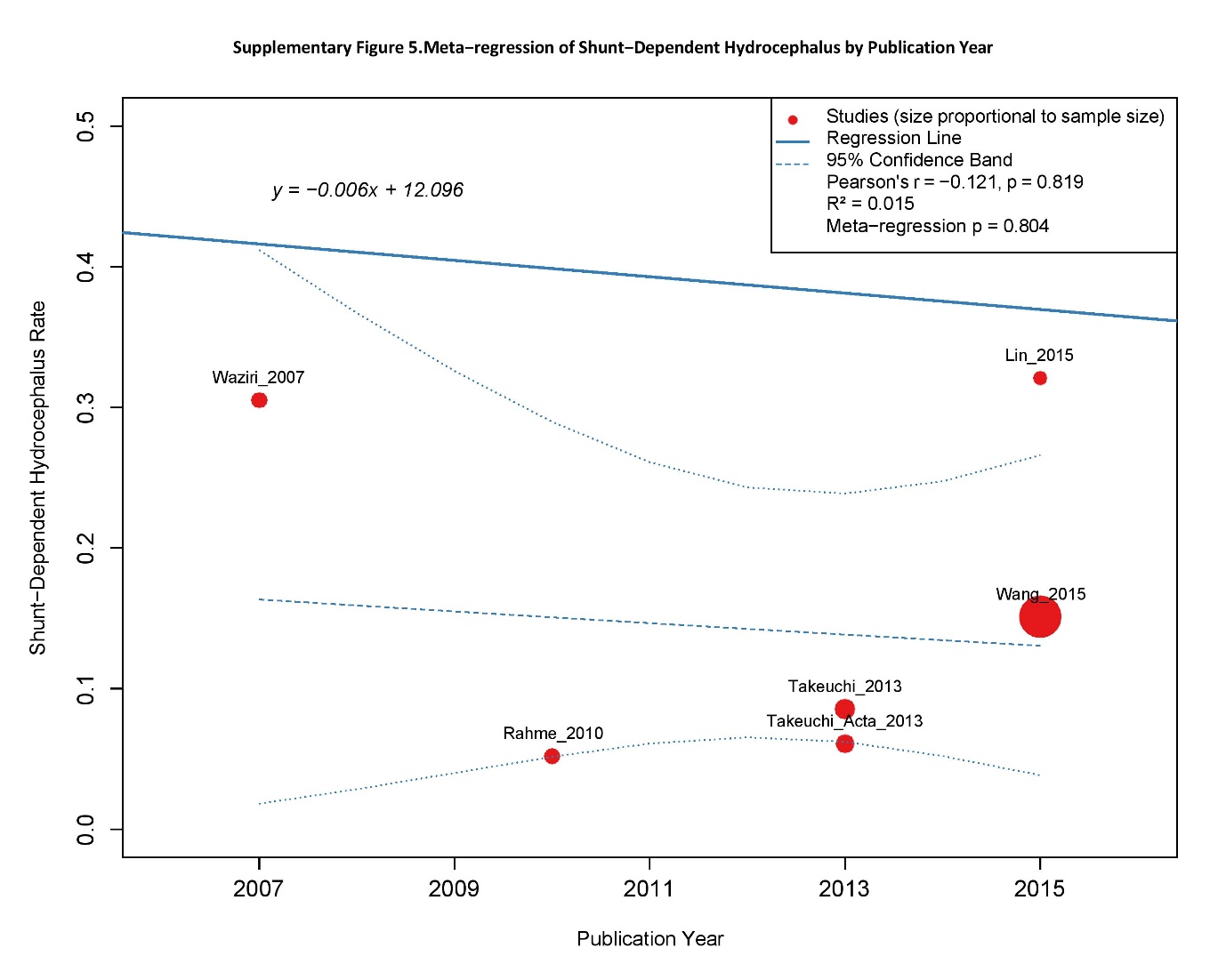


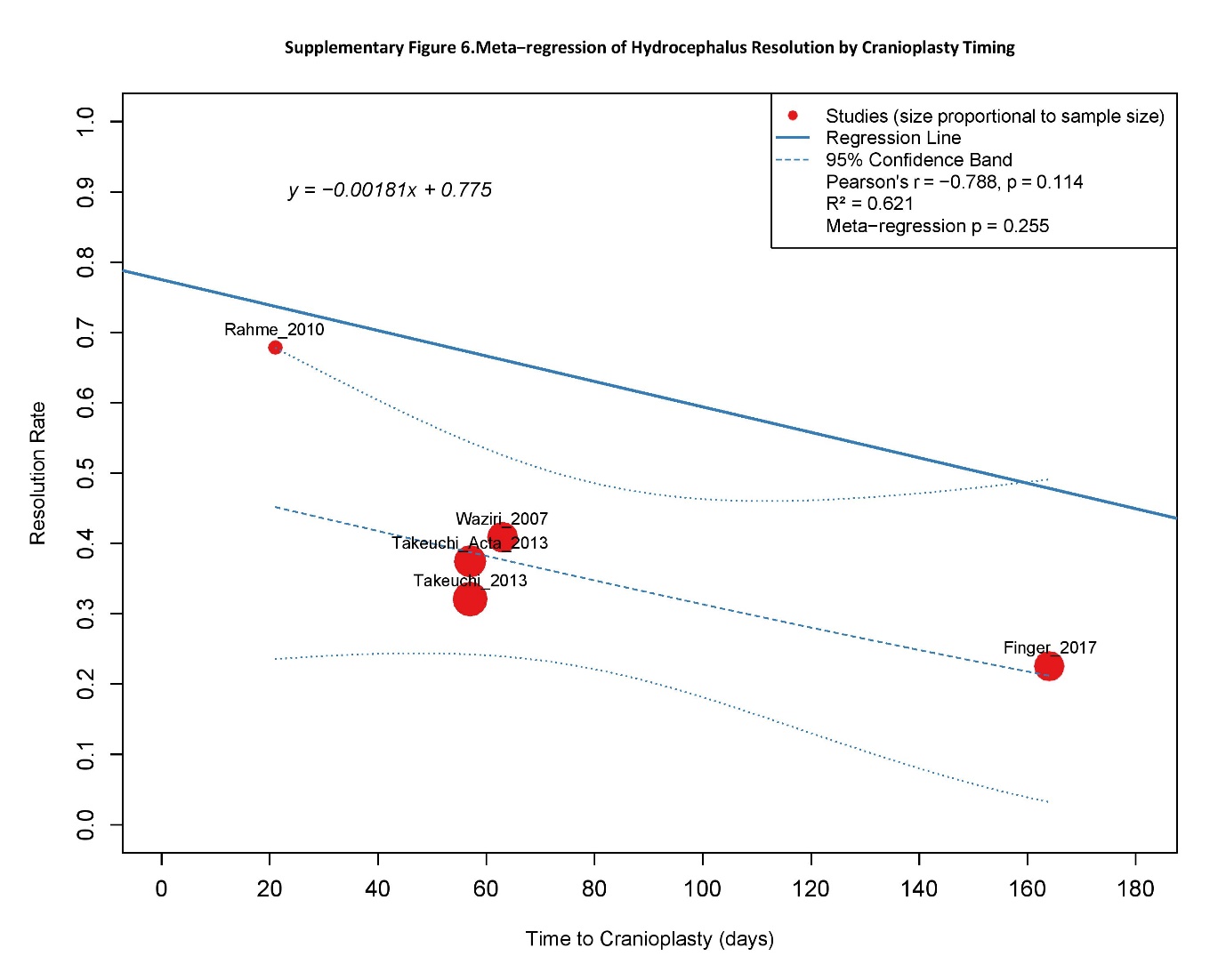


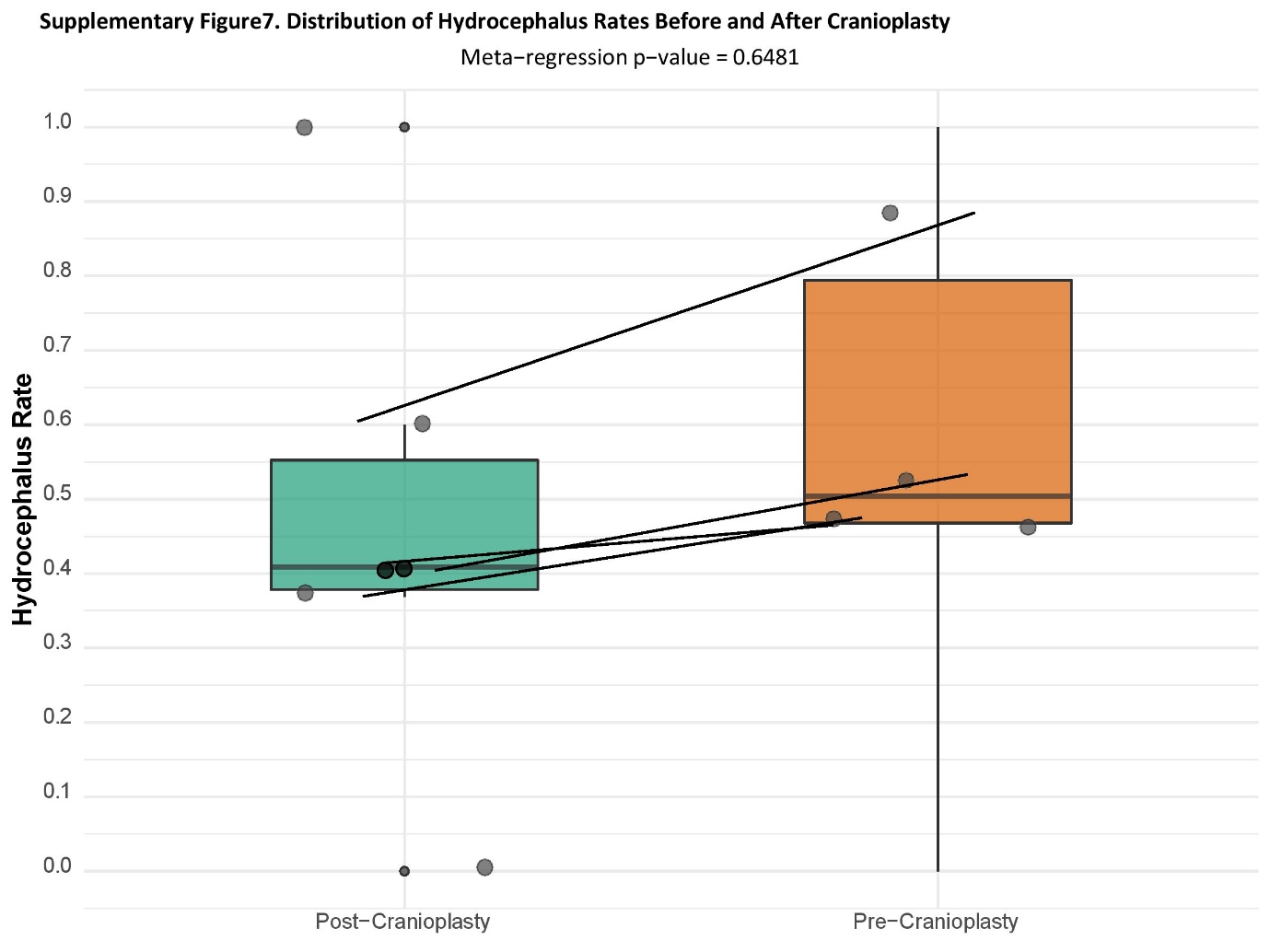


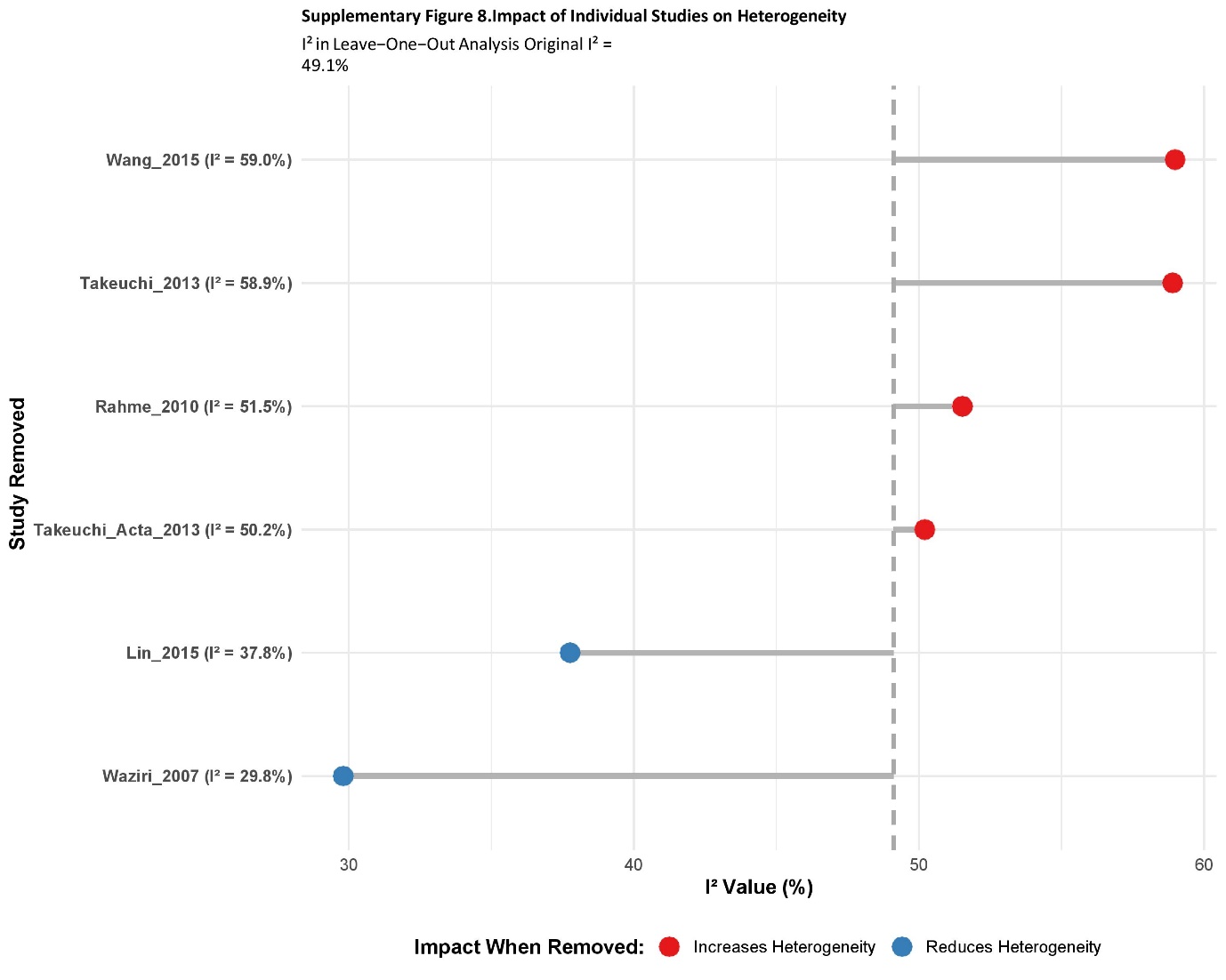


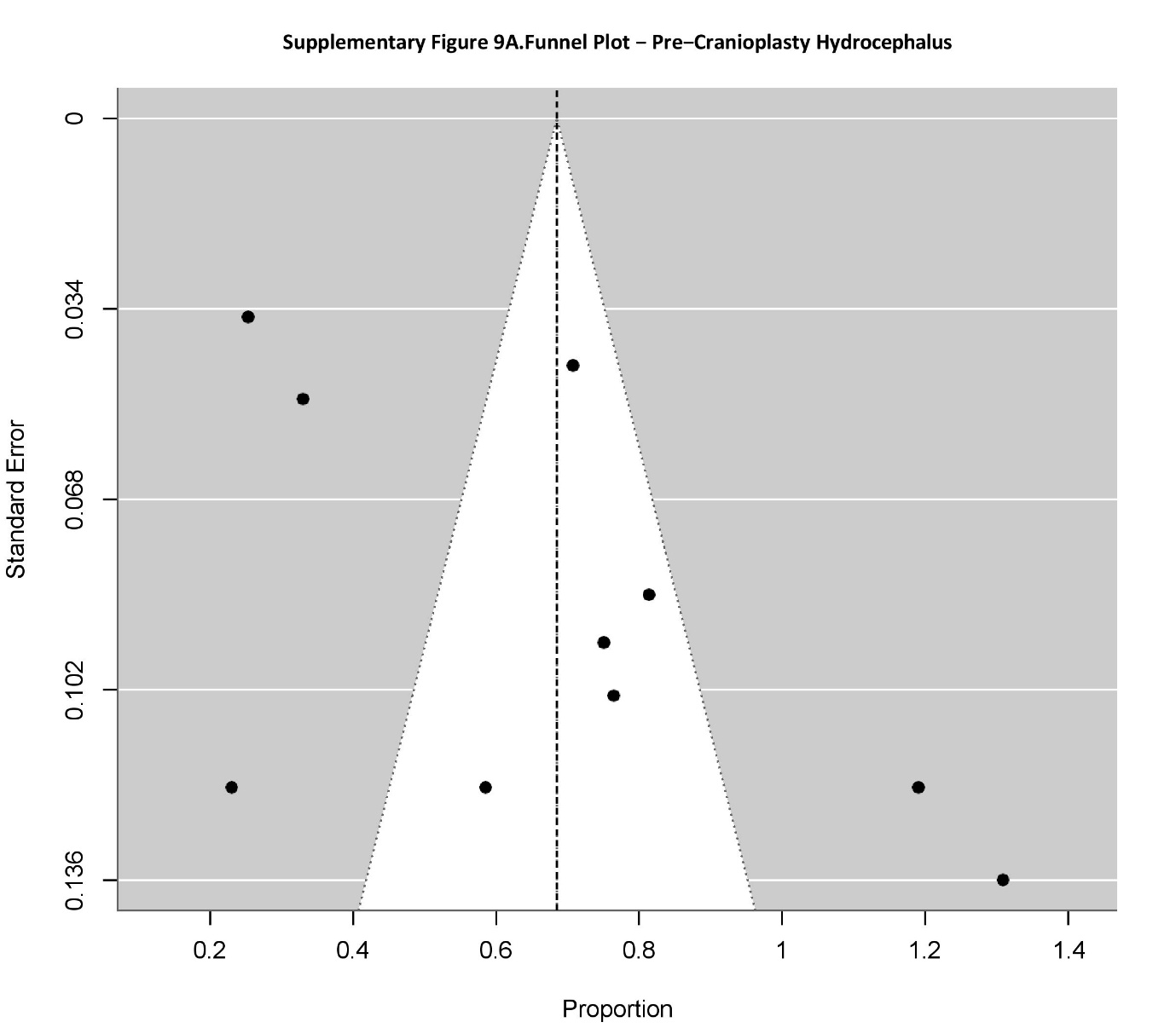


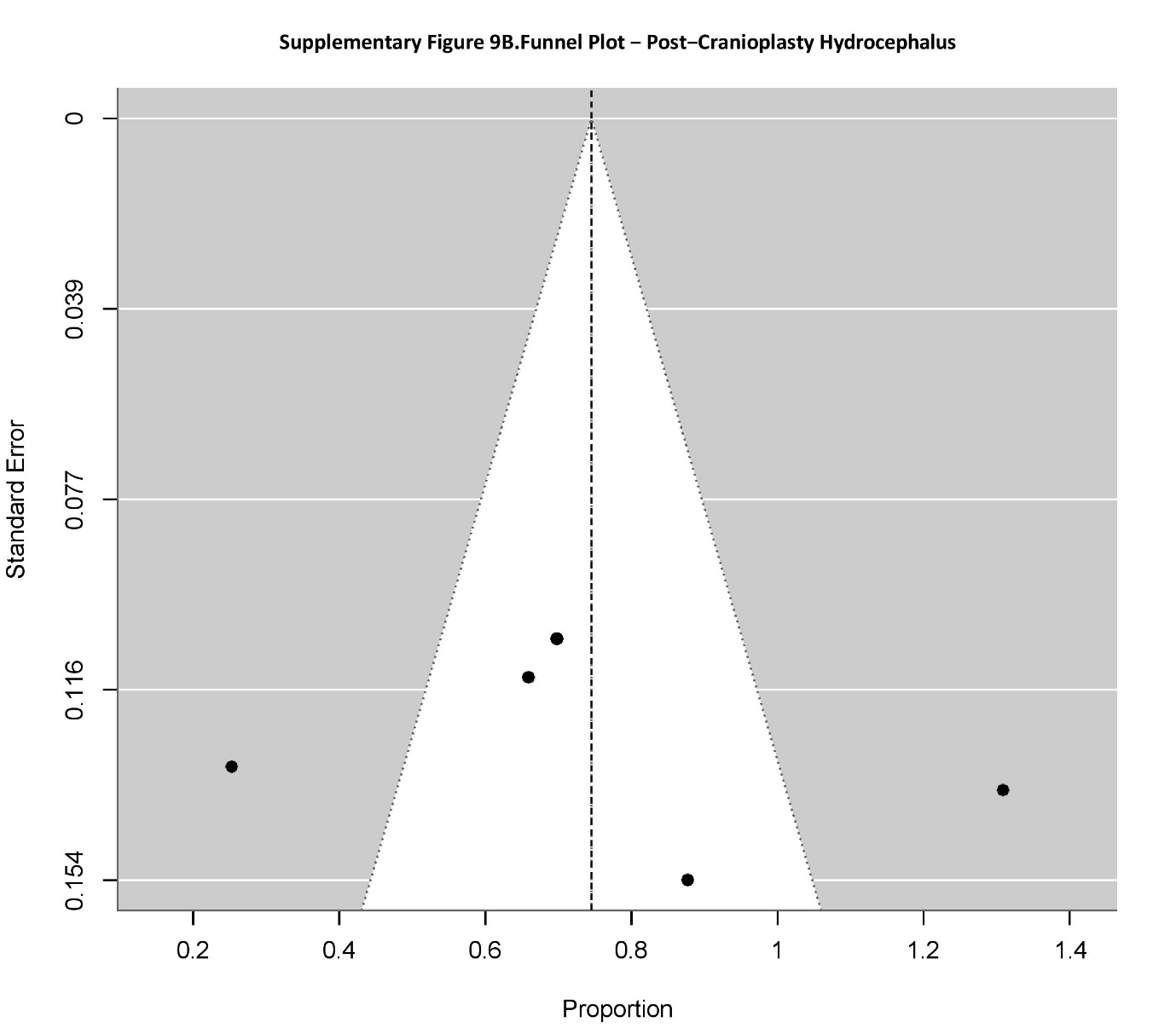

Supplement: Supplementary file 2 — Supplementary Material 2: Detailed results of sensitivity analyses (leave-one-out), meta-regression analyses exploring heterogeneity sources (publication year, cranioplasty timing), visual comparison of pre- and post-cranioplasty hydrocephalus distributions (box plot), and funnel plots assessing publication bias are provided in Supplementary Figs. 1–9. [file 10143_2025_3650_MOESM2_ESM.docx]
